# Effect of Compositionally Different Substrates on Elemental Properties of Bay Bolete Mushrooms: Case Study of 34 Essential and Non-essential Elements from Six Areas Affected Differently by Industrial Pollution

**DOI:** 10.1007/s12011-024-04429-5

**Published:** 2024-10-31

**Authors:** Alexandre V. Andronikov, Irina E. Andronikova, Ondrej Sebek, Eva Martinkova, Marketa Stepanova, Oksana Perehon

**Affiliations:** Division of Geochemistry and Laboratories, Czech Geological Survey, Geologicka 6, 15200 Prague, Czech Republic

**Keywords:** Bedrock, Mushroom, Soil, Trace elements, Translocation, Uptake

## Abstract

We studied concentrations of 34 essential and non-essential elements in samples of edible Bay Bolete (*Imleria badia*) mushrooms added by samples of the growing substrate and bioavailable fraction. The samples were collected from six forested sites affected differently by industrial pollution and underlain by compositionally contrasting bedrock: granite, amphibolite, and peridotite. In all cases, mushrooms behaved as a bioconcentrating system for elements such as Ag, K, P, Rb, S, and Se (BCF > 1) being a bioexcluding system for the rest of the elements analyzed (BCF < 1). Most analyzed elements displayed moderate to high within-mushroom mobility being accumulated preferably in the apical parts of the mushroom’s fruiting body (TF > 1). The highest mobility was demonstrated by Cd and Cu. Sodium was the only element with significantly low mobility (TF < 1), and it accumulated preferably in the stipe. *Imleria badia* seems to be sensitive to the accumulation of elements such as As, Cd, and Pb from the atmospheric deposits. Specific geochemistry of the growing substrate was reflected to different extend in the accumulation of elements such as Ag, Cu, Rb, S, Al, Ca, Fe, Ba, and Na in the mushroom’s fruiting bodies.

**Supplementary Information:**

The online version contains supplementary material available at 10.1007/s12011-024-04429-5.

## Introduction

Edible mushrooms are an important link in the human food chain, and they are actively collected and consumed in different countries worldwide. Mushrooms are known to effectively incorporate various elements from the growing substrate and, in particular, toxic elements such as As, Cd, Cr, Hg, Pb, and Se (e.g., [1–5]). Several studies conducted so far have suggested site dependency of the element uptake, i.e., soil composition and the extent of the soil pollution are supposed to be important factors affecting the chemical composition of mushrooms [2, 6–12]. If this suggestion is correct, mushrooms collected from polluted substrates can impose danger to human health if they are consumed in significant amounts. Based on the mushrooms’ possible affinity to changes in growing substrate composition resulting from industrial pollution by, in particular, trace metals, it was suggested that wildly growing mushrooms can be a good tool to monitor environmental pollution by both heavy metals [e.g., 13–17 and references therein] and radionuclides [e.g., 18–22 and references therein].

In spite of the shown similarity in mushrooms overall behavior with respect to uptake and accumulation of different elements (e.g., [8, 10, 23–25]), several works suggested that mushrooms of different species (both edible and non-edible) could display different behavior during uptake, translocation, and accumulation of the elements [26–29]. Since mushrooms grow readily in areas both unpolluted and significantly polluted [e.g., 11, 24, 30, 31], there is a good chance that mushrooms would respond differently to different concentrations of the polluting elements in the growing substrates. The Czech Republic is a country where closely located areas were/are contaminated differently by products of modern industrial activity [32–36].

Although a general agreement exists that mushrooms could be effective accumulators of varying elements including heavy metals and other toxic elements (e.g., [1, 3, 18, 28, 37]), it is not known much about the behavior of mushrooms, especially edible, with respect to certain compositional changes of the growing substrate [e.g., 23, 24, 28, 37, 38, 39]. It was suggested by [40] that Boletaceae mushrooms such as, for example, *Xerocomellus chrysenteron*, growing on compositionally contrasting substrates were not site-dependent, and only several elements reflected specific geochemistry of the substrate. However, that study was conducted on soils not affected by modern industrial pollution, and concentrations of heavy metals and other toxic elements were not high in the growing substrates. A study by [41] conducted on the *I. badia* samples grown on both unpolluted and significantly polluted substrates showed that the mushroom’s fruiting body may respond to the change in the growing substrate composition caused by the pollution. The extension of the study of the elemental composition of mushrooms and related substrates to the areas differently affected by industrial pollution and underlain by the contrasting bedrock may shed new light on the processes of element accumulation and translocation in the mushrooms’ fruiting bodies, and help to assess risks related to consumption of edible mushrooms growing in the polluted areas.

**Fig. 1:**
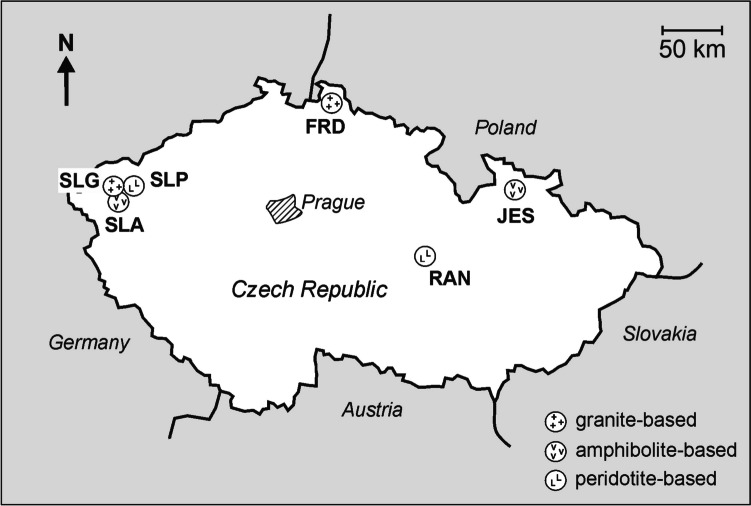
Schematic map showing location of the studied sites and compositions of the bedrock underlying the sites

In order to get insight into the behavior of various elements and minerals in mushrooms, it is necessary to examine fruiting bodies collected from the natural environment and assist with the examination of the underlain substrate [e.g., 23, 24, 28, 37, 39]. For the present study, we hypothesized that the pollution degree can significantly affect both the bioconcentration of elements by mushrooms and the within-mushroom distribution of the elements. In order to check the hypothesis, we studied samples of Bay Bolete (*Imleria badia*), one of the most highly picked and consumed mushroom species in the Czech Republic [42, 43]. All samples were collected almost simultaneously (within a four days span in the second half of August 2023) from six sites across the country (Fig. 1). The study sites are underlain by contrasting bedrock and were presumably affected differently by the modern industrial pollution [32–36, 44–49]. Several previous studies of *I. badia* (also known as *Boletus badius*, *Xerocomus badius*) from different areas were inconclusive and reported different behavior of individual samples. For example, Kojta et al. [41] showed that the mushrooms from significantly polluted areas accumulated more Ag, Pb, and Ba than the mushrooms from unpolluted areas, i.e., the mushrooms showed clear site dependency. On the contrary, Mleczek et al. [50] concluded that there were no significant differences in trace element compositions of the *I. badia* samples collected from different sites, and the difference in element accumulation depended on the particular year of sampling. Sotek et al. [51] suggested that the uptake of different elements by the *I. badia* mushroom may be influenced by different factors including in particular bioavailability and the total forms of elements in the growing substrate. In view of the features observed by different authors, we aimed to study the following: (i) to reveal whether the bioconcentrating potential of *I. badia* depends on the composition of (a) growing substrates and (b) on different degrees of the industrial pollution of the substrate; (ii) to reveal a proportion of elements in the fraction ready for fungi to uptake (bioavailable fraction); and (iii) to figure out whether uptake from the soil or accumulation from air deposit or both were mostly responsible for the presence of various polluting elements in the fruiting bodies of the *I. badia.*

## Materials and Methods

### Study Sites

Samples of the mushrooms and substrate soils were collected from six sites underlain by different bedrock: granite, amphibolite, and peridotite, across the Czech Republic (Fig. 1). Three sites (SLG, granite-based; SLA, amphibolite-based; and SLP, peridotite-based) are located in a very insignificantly polluted western part of the Czech Republic [34, 48]. Three other sites (FRD, granite-based; JES, amphibolite-based; and RAN, peridotite-based) are located in the northern (Jizera Mts., and High Ash Mts.) and central (Ransko Massif) parts of the Czech Republic, respectively, and were presumably significantly affected by the industrial pollution [32–34, 36]. Among the polluted sites, the influence of the industrial facilities located in southern Poland and in the Ostrava region of the Czech Republic could contribute to the FRD and JES sites pollution, respectively [32, 33, 35, 36, 47–49]. The RAN site was affected by ore mining from the seventeenth century until the 1960s that resulted in significant soil contamination by heavy metals which legacy pollution is detected until now [44–46]. Detailed characteristics of the areas studied can be found in [44–58].

### Sampling and Sample Preparations

We collected samples of the *I. badia* and the related substrate/soil samples (from the depth from 0 to 10–12 cm where the most mycelium is present affecting the substrate to the most [26]) in coniferous forests dominated by the Norway spruce (*Picea abies*). All samples (mushroom and soil) were collected from the areas both affected and not affected (or very insignificantly affected) by the industrial pollution, and from each identified substrate type (granite-, amphibolite-, and peridotite-based). For mushroom sample preparation, we applied techniques described in detail in, e.g., [19, 40, 59, 60] (see Appendix I in the Electronic Supplement for details). Bulk substrate soil samples were first air-dried for a few days and then sieved to a < 2 mm fraction. The sieved fraction was ashed at 550 °C for 8 h before processing and then homogenized in an agate mortar and quantitatively dissolved in an HF–HClO_4_ mixture. The samples dissolved in the HF–HClO_4_ mixture were dried down and then diluted in a mixture of “concentrated Ultrapure Romil HNO_3_—Milli-Q H_2_O” to 50 mL of 0.3N HNO_3_-based sample solution. To assess the bioavailability of the elements in the soil, we conducted a single-step extraction of bioavailable fraction according to the operational procedure accepted by our Institution [61]. Ten grams of the air-dried and sieved < 2 mm soil material were put into the acid-cleaned 100-mL polypropylene bottle, and 50 mL of 0.1 M BaCl_2_ extraction solution was added. The samples were shaken with a multi-functional Orbital shaker PSU-20i for 2 h. Thereafter, 25 mL more of 0.1 M BaCl_2_ extraction solution was added to each sample, and the solutions were centrifuged at 5000 rpm for 10 min at the Megafuge ST Plus Series centrifuge. Centrifuged sample solutions filtered through 0.45 µm PTFE syringe filters were added with 25 mL 0.1 M BaCl_2_ extraction solution and with 1 mL of 6 M HCl (up to 100 mL volume total).

### Analyses

Elemental compositions of all samples were determined with the Agilent Technologies 5110 inductively coupled plasma-optical emission spectrometer (ICP-OES) according to the standard operation procedure accepted by our laboratory [40]. In brief, samples in 0.3N HNO_3_–based solution were introduced to plasma through the Meinhard concentric glass nebulizer with the use of the Agilent Technologies SPS-4 autosampler. Samples of bioavailable fraction were analyzed in 0.1 M BaCl_2_ extract solutions. Altogether, 21 individual samples of mushrooms (each of the seven mushrooms’ fruiting bodies was divided into three subsamples: the stipe, the cap, and the sporophore) and seven related soil samples (added with seven samples of the bioavailable fraction) were analyzed for concentrations of 34 minor and trace elements (for details of the analytical technique see Appendix I in the Electronic Supplement). For quality control, we repeatedly analyzed procedural blanks and standard reference materials (SRM) such as SRM NIST 1515 (apple leaves), SRM NIST 2709a (San Joaquin soil), and SRM NIST 2711a (Montana soil) in batches with unknown samples. Three replicate analyses per sample provided us with an analytical error below 1% RSD for all determinations. Analytical results for the SRMs applied are given in the Electronic Supplement Table S1. All sample preparation and analytical works were conducted in laboratories of the Czech Geological Survey (Prague). Calculations of the Pearson correlation coefficients were done with the use of the Pearson Correlation Coefficient Calculator freely available online at https://www.socscistatistics.com/tests/pearson/default2.aspx.

The behavior of mushrooms with respect to uptake and accumulation of the elements was estimated via the bioconcentration factor (BCF). The BCF provides information on accumulating abilities of a system for any certain element [e.g., 12, 23, 51, 60], and it is expressed as follows:

1 $$\text{BCF}={C}_{\text{M}}/{C}_{\text{S}}$$

where *C*_M_ is the element’s concentration in the certain mushroom fruiting body part or in the mean fruiting body, and *C*_S_ is the element’s concentration in the growing substrate. We followed recommendations by [16] to estimate the degree of element accumulation: if BCF ≤ 0.01, no accumulation occurs, if BCF ≤ 0.10, a low degree of accumulation occurs, if BCF ≤ 1.00, a medium degree of accumulation occurs, and if BCF > 1.00, high degree of accumulation occurs (i.e., a mushroom can be considered an accumulating system).

Within-mushroom translocation of elements was expressed through the translocation factor (TF) which defines how mobile the element is. The TF determines the significance of relative translocation of the elements from soil (or parts of the mushroom) to other mushroom’s fruiting body parts [e.g., 62]. The TF is expressed as follows:

2 $$\text{TF}={C}_{\text{cap}}/{C}_{\text{stripe}}\left({C}_{\text{spor}}/{C}_{\text{cap}}\right)$$

where *C*_spor_ is the element’s concentration in the sporophore, *C*_cap_ is the element’s concentration in the cap, and *C*_stipe_ is the element’s concentration in the stipe. If the TF > 1, then the element displays high relative mobility.

## Results

Samples of the mushrooms, the corresponding growing substrate, and the soil bioavailable fraction displayed a wide range of compositions. Concentrations of elements in the bioavailable fraction have been always significantly lower than in both soil and mushrooms, but elemental relations between the mushrooms, bulk soil, and bioavailable fraction were not straightforward. The compositions of mushroom and bulk soil samples and the bioavailable fraction from the studied sites are given in Tables 1 and 2 and in the Electronic Supplement Table S3.

**Table 1:** Elemental composition of soil and bioavailable fraction samples (mg kg^−1^)

			Granite-based		Amphibolite-based		Peridotite-based			
			FRD	SLG	JES	SLA	RAN	RAN*	SLP	
Soil	Ag		0.23	0.98	0.18	1.28	0.18	0.11	0.21	
	Al		43,365	43,864	53,550	55,757	40,468	55,882	39,384	
	As		14.4	5.61	11.6	37.6	37.8	5.97	9.20	
	Ba		179	41.2	211	309	285	113	209	
	Ca		2592	1221	7985	15,891	1848	2631	14,269	
	Cd		0.25	0.10	0.54	0.95	0.83	1.81	0.42	
	Co		4.66	1.44	16.0	37.7	27.6	137	15.0	
	Cr		14.9	4.42	53.7	122	82.0	735	227	
	Cu		5.09	3.13	15.5	45.5	7.84	70.5	8.78	
	Fe		10,493	6365	36,671	36,580	29,413	109,831	24,809	
	Ga		16.0	18.8	15.5	17.4	12.8	20.7	10.8	
	K		18,004	21,477	9615	8104	11,189	4698	8693	
	Li		20.9	520	36.1	57.2	45.6	75.9	31.5	
	Mg		1269	645	9818	17,041	6046	15,225	21,635	
	Mn		80.5	136	575	625	1730	1331	452	
	Mo		0.75	bdl	bdl	bdl	bdl	bdl	bdl	
	Na		6811	4385	5548	6262	4582	2107	6458	
	Nb		19.3	14.9	14.4	8.91	11.7	6.49	7.47	
	Ni		3.50	1.60	18.3	55.2	156	920	127	
	P		468	329	429	547	267	264	223	
	Pb		153	121	26.1	41.7	60.1	22.3	26.4	
	Rb		242	559	94.8	97.8	149	55.6	113	
	S		234	122	227	403	188	218	236	
	Sb		bdl	bdl	bdl	bdl	bdl	7.07	2.20	
	Se		bdl	bdl	bdl	bdl	bdl	bdl	bdl	
	Sn		7.03	31.1	1.76	2.84	1.97	2.04	1.53	
	Sr		38.4	17.8	74.0	72.8	53.6	26.5	69.7	
	Ta		bdl	2.29	bdl	bdl	3.27	0.82	bdl	
	Ti		2408	1588	4971	4161	3017	5088	2829	
	V		33.6	7.04	88.7	119	51.3	110	61.6	
	W		6.30	8.80	1.16	2.01	1.63	1.03	1.45	
	Y		7.01	1.57	8.93	7.71	7.76	9.59	7.34	
	Zn		16.5	38.8	64.2	132	61.6	103	101.3	
	Zr		56.1	16.8	15.6	20.3	41.4	21.9	29.2	
Bioavailable fraction	Ag	bdl		bdl	0.036	bdl	0.037	bdl		bdl
	Al	481		285	461	447	31.7	112		230
	As	bdl		bdl	bdl	bdl	bdl	bdl		bdl
	Ba	n.d		n.d	n.d	n.d	n.d	n.d		n.d
	Ca	21.8		13.8	59.6	246	182	280		363
	Cd	0.08		0.03	0.04	0.09	0.16	0.08		0.05
	Co	0.02		0.01	0.15	0.53	0.14	2.94		0.71
	Cr	0.02		0.07	0.04	0.04	0.01	0.03		0.23
	Cu	1.00		0.74	1.05	0.72	0.62	0.86		0.69
	Fe	55.9		15.9	14.6	26.3	0.91	2.61		53.3
	Ga	bdl		bdl	bdl	bdl	bdl	bdl		bdl
	K	65.1		107	80.6	191	56.4	133		194
	Li	bdl		1.48	0.05	0.11	0.01	0.44		0.07
	Mg	12.3		8.81	20.7	52.9	1003	1659		241
	Mn	0.684		0.64	1.63	39.1	47.7	58.8		30.0
	Mo	bdl		bdl	bdl	bdl	bdl	bdl		bdl
	Na	36.3		19.0	33.8	31.1	25.3	30.7		34.2
	Nb	bdl		bdl	bdl	bdl	bdl	bdl		bdl
	Ni	0.18		0.16	0.15	0.66	14.1	24.1		5.32
	P	0.88		3.66	0.54	0.60	0.50	0.34		2.05
	Pb	5.02		6.35	1.82	1.74	0.40	0.80		2.06
	Rb	0.07		2.84	0.06	0.20	0.42	0.17		0.05
	S	3.72		3.93	3.47	6.48	0.90	2.00		7.15
	Sb	bdl		bdl	bdl	bdl	bdl	bdl		bdl
	Se	bdl		bdl	bdl	bdl	bdl	bdl		bdl
	Sn	bdl		bdl	bdl	bdl	bdl	bdl		bdl
	Sr	0.15		0.47	0.32	1.85	1.07	2.59		1.88
	Ta	0.30		bdl	bdl	0.13	bdl	bdl		bdl
	Ti	0.05		0.03	0.07	0.09	bdl	bdl		0.05
	V	0.46		0.10	0.48	0.96	0.12	0.03		0.04
	W	0.39		0.13	bdl	bdl	bdl	bdl		bdl
	Y	0.06		0.08	0.14	0.04	0.03	0.33		0.06
	Zn	0.90		1.43	1.01	3.00	2.81	3.16		4.00
	Zr	bdl		bdl	bdl	bdl	bdl	bdl		bdl

Detection limits in solutions for As, P, Pb, S, Sb, Se, Sn, and W were 0.15–0.35 mg L^−1^; for Co, Ga, Mo, Ni, and Rb were 0.05–0.08 mg L^−1^; for Ag, Al, Cd, Cr, Cu, Fe, K, Mn, Na, Nb, Ti, V, Y, Zn, and Zr were 0.01–0.03 mg L^−1^; for Ba, Ca, Li, Mg, Sr were 0.001–0.003 mg L^−1^

*bdl* below the detection limit, *n.d.* no data available

**Table 2:** Elemental composition of the *Imleria badia* fruting bodies (mg kg^−1^ dry matter)

	Granite-based		Amphibolite-based		Peridotite-based		
	FRD	SLG	JES	SLA	RAN	RAN*	SLP
Ag	1.52	0.83	0.39	1.61	0.63	0.74	0.99
Al	15.4	15.0	33.2	37.0	5.70	10.5	21.3
As	0.55	0.79	0.58	1.40	0.54	0.77	1.48
Ba	0.34	0.23	0.68	1.23	0.14	0.21	0.47
Ca	216	182	186	616	126	96.4	399
Cd	11.9	1.90	0.21	0.29	1.54	0.72	3.31
Co	bdl	bdl	bdl	bdl	bdl	bdl	0.14
Cr	0.39	0.13	0.17	0.23	0.12	0.11	0.33
Cu	34.3	20.3	23.3	30.0	14.7	34.5	27.6
Fe	23.6	24.4	44.8	44.7	19.4	22.0	34.6
Ga	bdl	bdl	bdl	bdl	bdl	bdl	bdl
K	19,020	21,662	21,362	18,888	16,236	24,856	15,694
Li	bdl	bdl	0.11	bdl	bdl	bdl	bdl
Mg	540	630	605	663	477	605.1	765
Mn	3.32	8.74	12.1	20.1	4.11	4.92	6.95
Mo	0.21	0.18	0.14	0.17	0.15	0.19	bdl
Na	1013	719	927	984	909	860	1151
Nb	0.04	0.10	0.04	0.06	0.09	0.09	0.43
Ni	0.31	0.46	0.35	0.84	0.44	0.64	2.00
P	3512	4828	3604	5274	3156	3286	6111
Pb	bdl	bdl	bdl	bdl	bdl	bdl	bdl
Rb	1021	1958	607	746	503	293	538
S	4127	3038	2429	4273	3356	3646	6886
Sb	bdl	bdl	bdl	bdl	bdl	bdl	bdl
Se	0.63	0.76	0.82	0.65	0.87	0.61	10.9
Sn	0.38	0.43	0.32	0.42	0.35	0.51	0.45
Sr	0.46	0.54	0.76	1.32	0.18	0.41	0.83
Ta	bdl	bdl	bdl	bdl	bdl	bdl	bdl
Ti	0.10	0.14	1.20	1.13	0.12	0.14	0.65
V	bdl	0.04	0.14	0.06	bdl	0.07	bdl
W	1.05	0.90	0.86	1.01	0.54	0.88	1.08
Y	bdl	bdl	bdl	bdl	bdl	bdl	bdl
Zn	121	113	97.4	118	61.2	104	124
Zr	bdl	bdl	bdl	bdl	bdl	bdl	bdl

Composition of the fruiting bodies was calculated from the relative amount of individual ruiting body parts (see Table S3 in the Electronic Supplement)

*bdl* below the detection limit of the method

### Trace Element Characteristic of the Soils

The soils developed on the bedrock of the three identified types expectedly displayed different elemental compositions regardless of the site pollution degree (Table 1). The peridotite-based soils from both SLP and RAN sites displayed much higher concentrations of elements such as Co, Cr, Mg, and Ni than the granite- and amphibolite-based soils. Amphibolite- and peridotite-based soils were both characteristically richer in major elements such as Al, Ca, Fe, and Na, and in trace elements such as Cd, Mn, Sr, V, and Zn than the granite-based soils. The amphibolite-based soils from the SLA and JES sites were enriched in As, Ba, and Ti compared to both granite- and peridotite-based soils. The granite-based soils were typically poorer in most analyzed elements with the exception of higher concentrations of K, Li, Pb, and Rb, which concentrations are higher than in both amphibolite- and peridotite-based soils.

Some differences in elemental compositions were observed for the soils from different locations but developed on bedrock of the same type. Although granite-based soils from the FRD and SLG locations displayed a lot of similarity with respect to most trace element concentrations, some trace elements were present in different amounts. Higher concentrations of elements such as Li and Rb in SLG samples were due to the specific compositions of the bedrock [56]. The same can be said about the higher concentrations of Ba, Ca, Mg, Sr, and Zr in soils from the FRD site [63]. However, much higher concentrations of generally atypical for granite elements such as Co, Cr, Ni, and V in soils from the FRD site could be due to contamination of soils with materials related to the industrial activity. But this aspect requires a much more detailed study.

Amphibolite-based soils from the JES and SLA sites displayed measurable differences in the concentration of various elements. Differences in concentration of the elements such as Ba, Ca, and Mg were due to the compositional differences of the corresponding bedrock [55, 64]. It is important to note that at the beginning of our study, we suggested that the JES site located very close to the Polish border should be significantly affected by industrial pollution [36]. However, it has occurred that the soils from the amphibolite-based JES site displayed much lower concentrations of the elements which are supposed to be introduced by the industrial activity (As, Co, Cr, Cu, Ni, V, and Zn) compared to the concentrations of these elements in the amphibolite-based SLA site, which was presumably almost free from the modern industrial pollution influence [48]. Therefore, the JES site should be considered as an example of an area with a very low pollution level.

Soils from the peridotite-based RAN site (known for mining activity from the seventeenth century until the 1960s) were collected from two different spots. The soils at the first spot (samples denominated as RAN) were collected under the spruce canopy away from old mines. The soil at the second spot (samples denominated as RAN*) was collected near the mouth of an old mine from the remnants of a mining waste pile. The soil from the peridotite-based SLP site was collected under the spruce canopy (with no mining activity in the region whatsoever). Soil samples from the RAN and SLP sites displayed differences in the concentration of some elements. Differences in concentration of the elements such as Ca, Cr, Mg, and Zr between the RAN (sensu lato) and the SLP samples were due to the compositional difference of the bedrock [45, 53, 65]. Differences in concentration of As and Pb (concentrations were higher in the RAN samples with similarly low concentrations in bedrock from both RAN and SLP sites) should be due to contamination of the RAN soils with materials resulting from the industrial activity. The RAN* soils displayed several compositional features differing them from the RAN soils. The difference was mostly pronounced in much higher concentrations of Cd, Co, Cr, Cu, Fe, Mg, Ni, Sb, Ti, V, and Zn in the RAN* samples that is definitely due to the delivery to the surface of the materials from Ni-Cu–Zn deposits developed in the region [45, 66]. Lower concentrations of Ba, K, Na, Rb, Sr, and Zr in the RAN* soils were also due to the difference in the composition of peridotite bedrock in the Ransko massif on the one hand and subsurface ore deposits rich in certain elements on the other hand [45, 66].

Four groups of the elements could be distinguished for the bioavailable fraction (extracted from bulk soil samples) based on the soil’s response to the extraction (Table S2 in the Electronic Supplement). Elements of the first group (i) always gave a very insignificant response to the extraction, and the amount of the elements extracted was always < 1% of the elements’ amount in the bulk soil sample (e.g., Al, Cr, Fe, Li, Rb). Elements of the second group (ii) always gave a significant response to the extraction, and the amount of the elements extracted was high, often > 10% of the amounts in the bulk soil sample (e.g., Cd, Cu, Ni, Zn). Elements of the third group (iii) displayed erratic response to the extraction with the amount of the elements extracted from bulk substrate samples varying from < 1% to sometimes > 10% of the amount of the elements in the soil (e.g., Ca, Co, Sr, Y). The fourth group (iv) is represented by the elements which amounts in the bioavailable fraction were very low, mostly below the detection limits of the analytical method (e.g., Ag, As, Sb, Zr) (the detection limits for the elements analyzed are given in the Electronic Supplement Table S3).

### Trace Element Characteristic of the Mushrooms’ Fruiting Bodies

Conducted analyses of the *I. badia* samples showed high variability in concentrations of different elements in different parts of the mushrooms’ fruiting bodies (Table S3 in the Electronic Supplement). The measured concentrations of different elements were overall similar to those reported for *I. badia* samples from elsewhere [e.g., 4, 41, 50, 51, 67]. The highest amounts were displayed by the essential elements such as Ca, Mg, Na, P, S, and Zn [3, 67] which concentrations in the fruiting bodies varied from hundreds to thousands mg kg^−1^ (here and further on, dry matter when we are talking about mushrooms). Most other analyzed elements (both essential and non-essential) were present in mushroom samples in lower amounts. Notably, some elements typical for the studied soil samples (Ga, Li, Sb, Ta, Zr), were not detected in mushroom samples, while others (e.g., Se) present in very low amounts (down to the bdl) in the soils studied were abundant in the mushroom samples.

Site-dependency was observed to varying extents for elements such as Ag, Cu, Rb, S, Al, Ca, Fe, Ba, and Na. Although such a list of the site-dependent elements is longer than that described by [40] for *X. chrysenteron* samples, most site-dependent elements were similar for both *X. chrysenteron* and *I. badia* samples. Mushroom samples from the granite-based sites SLG and FRD were expectedly more abundant in Rb (830–2830 mg kg^−1^ for different parts of the fruiting body) than samples from the amphibolite- and peridotite-based sites (235–1045 mg kg^−1^) consistently with higher concentrations of Rb in the granite-based soils (190–860 mg kg^−1^ in granite-based soils vs. 55–155 mg kg^−1^ in soils from the two other sites). Barium (0.5–1.6 mg kg^−1^), Fe (27–61 kg^−1^), Mn (11–32 mg kg^−1^), and Ti (0.8–1.6 mg kg^−1^) were more abundant in the mushrooms from the amphibolite-based sites JES and SLA than in the mushrooms collected from other localities. This overall corresponds to higher concentrations of Fe and Ti (but not Ba and Mn) measured in the amphibolite-based soil samples. On the contrary, mushroom samples from the two amphibolite-based localities displayed the lowest concentrations of Cd (0.07–0.4 mg kg^−1^) compared to mushroom samples from other localities (0.2–23 mg kg^−1^). It is notable that the highest concentrations of Cd (4.2–23 mg kg^−1^) were measured in mushroom samples from the granite-based FRD site whereas samples from the granite-based SLG site displayed significantly lower concentrations of the element (0.8–3.4 mg kg^−1^). Mushroom samples collected from the waste pile at the old mine mouth (peridotite-based RAN* spot) displayed lower concentrations of Cd (0.24–1.1 mg kg^−1^) than the mushrooms from the peridotite-based RAN and SLP sites (0.7–3.7 mg kg^−1^). Arsenic displayed higher concentrations in mushroom samples from the western part of the Czech Republic (SLA, SLG, and SLP sites) than in their counterparts from the northern and central areas of the country (FRD, JES, and both RAN sites). That is, samples from the granite-based substrate in the western part of the country were richer in As than samples from the granite-based substrate in the northern part of the country, etc. Selenium displayed higher concentrations in mushroom samples collected from the peridotite-based SLP site (9–11 mg kg^−1^) than from any other site studied (from the bdl up to 6.8 mg kg^−1^) although concentrations of the element were very low (bdl) in all soil samples analyzed [40]. Nickel concentrations were also much higher in mushroom samples collected from the peridotite-based SLP site (1.9–2.1 mg kg^−1^) than from any other sites (0.2–1.1 mg kg^−1^) including Ni-rich peridotite-based soils RAN and RAN*.

## Discussion

### Accumulation of the Elements in Mushroom Fruiting Bodies

Overall, mushrooms are known to display varying abilities to concentrate different trace elements [20, 37, 38, 68]. Samples of the *I. badia* analyzed were characterized by the different behavior of different elements. Mushrooms’ fruiting bodies as a whole always behaved as bioconcentrating systems with respect to the elements such as K, P, Rb, and S (BCF varied from 1.0 to 1.1 for K in mushroom samples collected from the granite-based SLG and FRD sites to 29 for S in mushroom sample from the peridotite-based SLP site) (Table S4 in the Electronic Supplement) which is not a surprise since these elements are bioessential [67]. The mushrooms studied have behaved mostly as bioconcentrating systems with respect to Ag (BCF = 1.2–9.9, except for a sample collected from the granite-based SLG site with the BCF = 0.86 that corresponds to a medium degree of accumulation only). It seems that Ag is one of the elements readily accumulating in Boletaceae mushrooms as a group and in the *I. badia* in particular [37, 40, 41, 59, 69]. The mushrooms studied can behave as either bioconcentrating or bioexcluding systems with respect to Cd, Cu, and Zn (BCF = 0.3–47). Among these three elements, the behavior of Cd is interesting. Although overall Cd concentrations in the samples analyzed were mostly within the range of values known for wild foraged mushrooms [e.g., 1, 4, 31], its concentrations significantly varied in samples studied here. Cadmium was enriched in mushrooms from the two granite-based sites (BCF = 19–47) where the element’s concentration in bulk mushroom samples varied from 1.9 to 12 mg kg^−1^ and in mushrooms collected from the peridotite-based sites RAN and SPL (BCF = 1.9–7.5) where the element’s concentration in bulk mushroom samples varied from 1.9 to 3.1 mg kg^−1^ vs 0.3 to 0.4 mg kg^−1^ in the soil. On the contrary, in samples collected from the two amphibolite-based sites and from the peridotite-based spot RAN* (at the old mine mouth), Cd behaved rather as a bioexcluded element (BCF = 0.40–0.83) although the concentration of the element could reach 1.8 mg kg^−1^ in the related soil. Other elements analyzed were bioexcluded showing the BCF values significantly below unity regardless of the sampling site and the soil type (Table S4 in the Electronic Supplement).

Similarly to samples of *B. edulis*, *X. chrysenteron*, and *I. badia* mushrooms from elsewhere [40, 51, 59, 70, 71] where the BCF for selenium was as high as 121 (*B. edulis* samples from NW Poland [51]), Se in all presently analyzed mushroom samples was enriched relative to the substrate (with concentrations of Se in all analyzed substrates always bdl). However, very high concentrations of Se (up to 11 mg kg^−1^, Table S3 in the Electronic Supplement, which concentrations are significantly higher than those reported for *I. badia* samples from elsewhere [51, 71]) were observed only once in a sample from the peridotite-based SLP site. Samples collected from other sites including the peridotite-based RAN site displayed much lower concentrations of Se (0.5–0.8 mg kg^−1^; Table S3 in the Electronic Supplement) comparable to those in *I. badia* samples from elsewhere [51, 71]. Such concentrations are overall lower than those reported by [40] for *X. chrysenteron* samples collected from the granite- and amphibolite-based sites (0.9–1.6 mg kg^−1^), and much lower than concentrations of Se measured in *B. edulis* samples collected from the amphibolite- and peridotite-based sites (7–32 mg kg^−1^; [59]). Significantly higher concentrations of Se in fungi than in the related substrate are a known phenomenon described in multiple publications [e.g., 51, 72 73, 74]. However, similarly significant uptake of the element by mushrooms of the two different species (*B. edulis*, which is expected, and *I. badia*, surprisingly) growing at the same site on the same substrate only (peridotite-based) compared to any other analyzed substrate is something new and requires more detailed studies to be explained.

We have figured out that the site-dependency was pronounced to a larger or smaller extent for several analyzed elements (Ag, Al, Ba, Ca, Cu, Fe, Rb, Na, and S) whereas others either did not display the site-dependency or displayed it to a limited extent only. Sodium, Cu, and Ag were the elements for which the site-dependency was pronounced to the best. Our data seem to be consistent with those reported by [41] who showed site-dependency of the *I. badia* with respect to elements such as Ag, Pb, and Ba. However, Kojta et al. [41] showed the site-dependency for strongly polluted environments only, whereas we demonstrated that the *I. badia* could depend on the composition of the growing substrate in much less polluted environments as well.

**Fig. 2:**
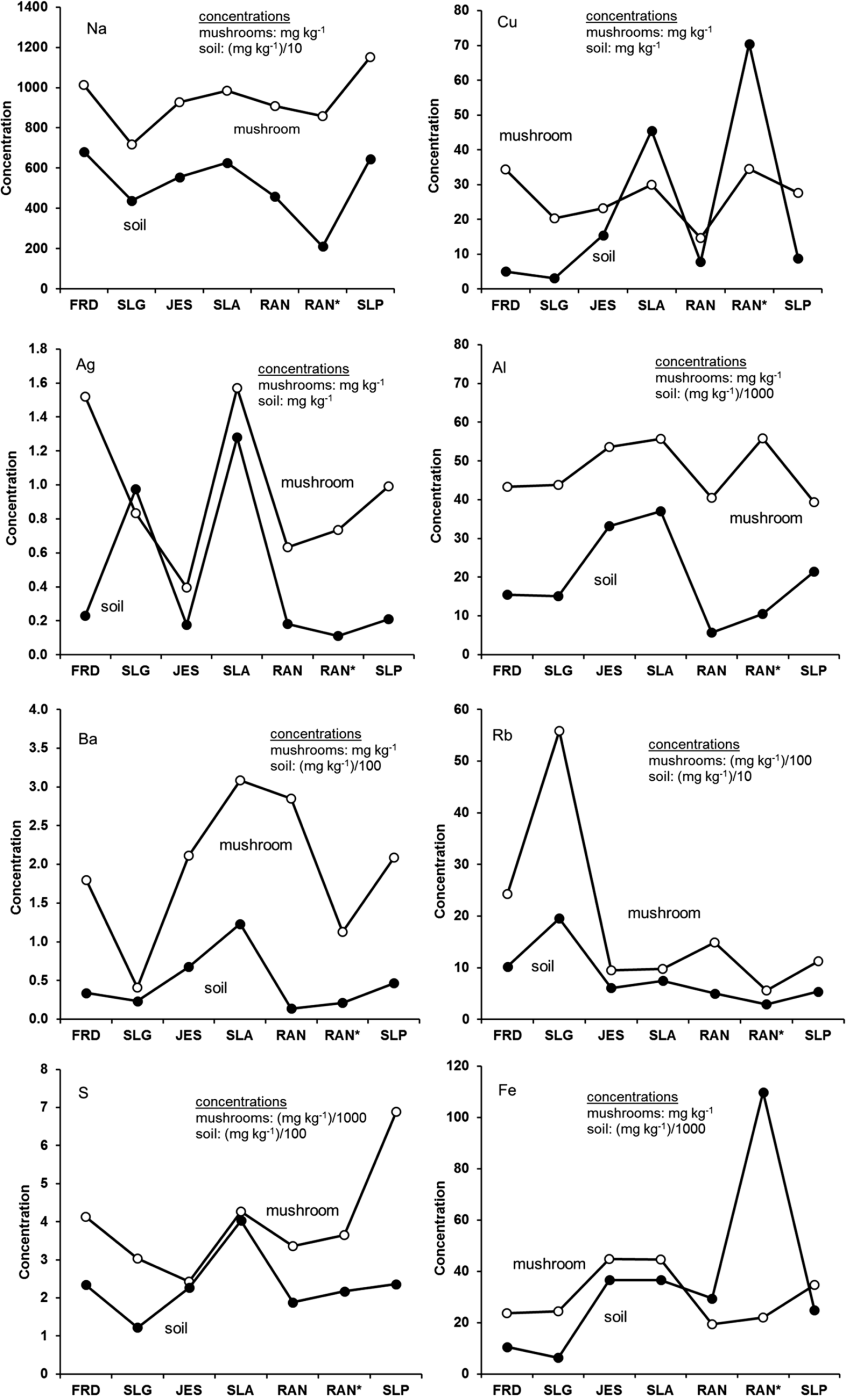

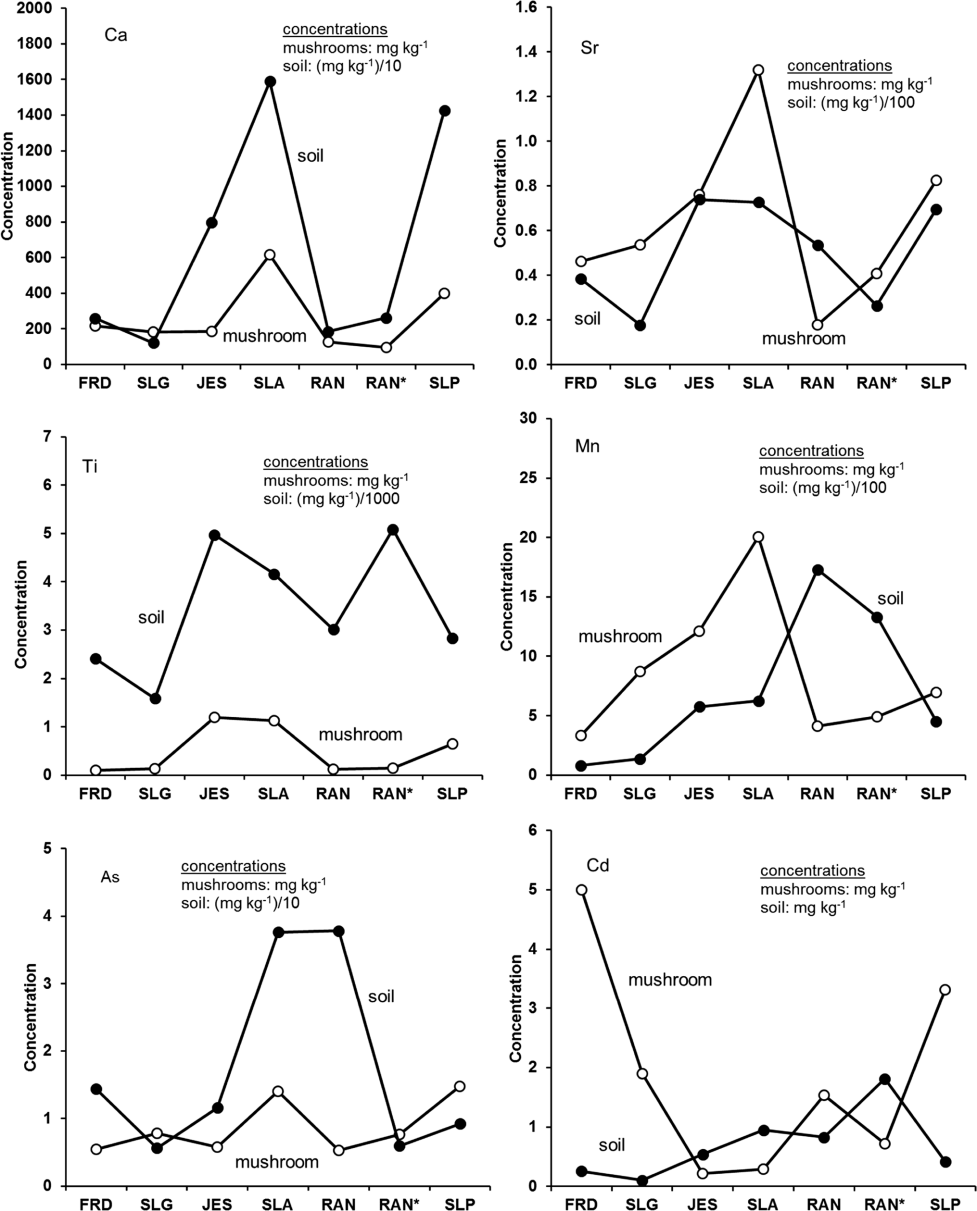
Diagrams showing relationship between the concentrations of the selected elements in the mushroom fruiting bodies and related substrates. See text for more details. Concentrations of most elements in soils are reduced for the sake of consistency. The lines connect individual data points for the sake of demonstrativeness

Selective site-dependency for some elements is demonstrated well in the diagrams in Fig. 2. Similarity in the behavior of the elements in both the mushrooms and the soil was pronounced the best by Na, Cu, and Ag. That is, the higher the concentration of the element in the soil was, the higher it was in the related mushroom. Overall, a positive correlation between concentrations of Na in the mushrooms and in the growing substance seen in Fig. 2 was insignificant (Pearson’s correlation coefficient *R* = 0.662 and *p* < 0.10), but if we omit samples from the SLG and SLP sites, the correlation becomes significant (*R* = 0.946 and *p* < 0.01). Concentrations of Cu in the analyzed mushroom samples were also site-dependent that is reflected in an insignificant positive correlation between the Cu concentrations for all mushroom samples and all bulk soil samples (*R* = 0.554 and *p* < 0.10). However, again, if we omit FRD and RAN samples, the correlation becomes significantly positive (*R* = 0.901 and *p* < 0.05). A similar style of the element’s relation between the mushroom and substrate samples was demonstrated by Ag (*R* = 0.759 and *p* < 0.10 for all sites except for FRD samples).

At the FRD site, very high concentrations of Ag in the mushroom were not consistent with significantly lowered element concentrations in the soil (Fig. 2). The reason for such strong enrichment of the mushrooms in Ag is questionable and needs further detailed consideration. Since there is a positive correlation between the concentrations of Ag in the substrate and in the mushroom for all sites except for the FRD, it seems that the extra amounts of Ag were not taken by the mushrooms from the substrate soil. It is reasonable therefore to suggest that extra amounts of Ag were delivered to the FRD mushrooms with atmospheric deposit. However, it is just a suggestion, and the reason(s) for such strong enrichment of mushrooms in Ag needs to be studied in more detail. In particular, since [41] pointed out that the *I. badia* mushrooms collected near a former gold and copper mining area displayed significantly elevated concentrations of Ag compared to samples collected from non-polluted sites, it is obvious that an extra amount of Ag can be delivered in mushrooms from the substrate at some conditions. Aluminum, Ba, Fe, Rb, and S displayed insignificant positive correlation between the mushroom and soil (*R* = 0.550 to 0.864 and *p* < 0.05 to < 0.10) suggesting site-dependency (not ideal though) for the elements. Although the correlation is seen for Ca, Sr, and Ti, on diagrams in Fig. 2, the correlation is not statistically supported. Manganese behaves somewhat unusual: it displays a positive correlation for mushroom and soil samples from the granite- (FRD, SLG) and amphibolite-based (JES, SLA) substrates, and a rather negative correlation in the case of samples from the peridotite-based substrates RAN and SLP (Fig. 2). Magnesium, Ni, and W did not show any response to the change of the substrate composition displaying very similar concentrations in all mushroom samples (Table 2, and Table S3 in the Electronic Supplement). The rest of the elements analyzed did not show site-dependency randomly changing concentrations in mushrooms regardless of the substrate composition. Very high concentrations of As in soils from the amphibolite-based SLA and peridotite-based RAN sites (Table 1) were not reflected in As concentrations of bulk mushrooms whatsoever (Table 2) making the element completely site-independent. There was a very significant Cd enrichment for the mushroom samples collected from the peridotite-based SLP and granite-based FRD sites (Table 2 and Table S3 in the Electronic Supplement). Since there are no signs of substrate enrichment in Cd at these two sites (Table 1), it is reasonable to suggest that extra amounts of the element were delivered to mushrooms not from the growing substrate, but rather with atmospheric deposit.

Much less significant variations in concentrations of individual analyzed elements in the mushrooms than in the soils suggest that although amounts of the elements in the fruiting body of the *I. badia* depend to some extent on the substrate composition, the mushroom uptake was mainly regulated by the element amounts necessary for the mushroom’s life circle [18, 26, 40, 75]. It follows from all the considered that the site-dependency is a phenomenon depending on multiple factors yet to be studied in detail.

### Within-Mushroom Translocation of the Elements

A study of the *I. badia* samples from Poland [76] showed different accumulation abilities for different elements by different parts of the mushroom’s fruiting body, i.e., different mobilities were observed for different elements. A detailed work by [25] about the distribution of trace elements within different parts of a sample of the *Neoboletus luridiformis* mushroom (101 analyses of partial subsamples) from Slovakia showed a very complex distribution of individual elements within the mushroom’s fruiting body favoring the division of the bulk fruiting body to three main subunits: the stipe, the cap, and the sporophore. Individual elements accumulate to different extents in different subunits because of different mobilities of different elements. In the present study, a significant number of the elements analyzed in the *I. badia* samples have displayed moderate to high mobility that was reflected in different TF values for different elements (Table S5 in the Electronic Supplement). The elements with moderate to high mobilities were mostly those described as mobile for various mycorrhizal fungi [2, 8, 24, 25, 40, 59, 77]. However, the present study showed that the list of such moderately mobile elements (Ag, Al, As, Ca, Cd, Cu, Fe, Mg, Mo, P, Se, W, and Zn; TF_cap/stipe_ = 1.1–2.9) is longer than it was described by [40, 59] for the *Boletus edulis* and *Xerocomellus chrysenteron* mushrooms collected from the SLA, SLG, and SLP sites. In the case of highly mobile elements (except for Mg in samples from the SLP site, and Ca, K, Rb, and Se from all sites: elements displayed moderate mobility accumulating preferably in the cap (TF_cap/stipe_ = 1.1–3.5, TF_spor/cap_ = 0.43–0.96)), sporophore was their main accumulator (TF_spor/cap_ = 1.1–4.2). In the presently studied samples of the *I. badia*, Cd and Cu demonstrated the highest mobility among all elements (TF_spor/cap_ up to 4.3 and 3.8, respectively) accumulating in the apical parts of the fruiting body. High mobility of these elements for several Boletaceae mushroom species (including *I. badia*) was described by [e.g., 4, 25, 40, 41, ]. Among all the elements, only Na displayed low mobility preferably accumulating in the stipes (TF_cap/stipe_ = 0.28–0.95) that is consistent with the data by [25] for the *N. luridiformis* mushroom. Other elements behaved erratically displaying various degrees of mobility.

The high mobility of the elements suggests their preferable accumulation in apical parts of the fruiting body. Since toxic elements such as As, Cd, and Cr demonstrated not only high but also sometimes very high mobilities (TF_spor/cap_ up to 4.3 for Cd), their accumulation in apical parts of the mushrooms could impose a higher risk for human health. Therefore, it is more advisable to collect and consume the mushroom stipes rather than caps and sporophores (regardless of the gastronomical properties of individual mushroom parts).

### Mushroom-to-Soil Interaction

In order to assess the availability and pools of the elements readily taken up by mushrooms, we extracted potentially bioavailable fractions from bulk soil samples. The applied single-step extraction procedure could simulate in the first approximation the process of fungal solubilization by releasing weak organic acids (e.g., [78–83]). The concentration of different elements in bioavailable fractions extracted from soil samples was different, and four groups of the elements were identified on the basis of their response to the extraction (see section above and Table 1 and Table S2 in the Electronic Supplement). It is obvious that the simulations applied were not comprehensive, and it would be necessary to apply various and much more sophisticated extraction techniques to explain the transfer of some elements from bulk soil to the bioavailable fraction (e.g., [26, 27, 30, 84–87]). In spite of significant simplification, experiments conducted during the present study can give some ideas about certain elements’ ability to be transformed from the soil to a form much easier for mushrooms to uptake.

A high amount of elements in the bioavailable fraction does not always suggest their effective uptake by mushrooms [26, 88] because mushrooms seem to be very selective in the elemental uptake [18, 26, 75]. It implies that the intensity of the uptake is mainly regulated by the mushroom itself [26–28]. In order to estimate quantitatively the ability of the *I. badia* mushrooms to uptake elements from the bioavailable fraction, we calculated amounts of the elements present in the mushroom samples to compare these amounts with those transferred to the bioavailable fraction from the related bulk soil samples. Based on the depth from which mycorrhizal mushroom mycelium mostly takes up elements and nutrients (i.e., where the most mycelium is present, 10–12 cm; [27]) and a specific gravity of soils of 2.60–2.80 g/cm^3^ [89], the weight of the soil affected by the interaction with the mushroom’s mycelium was calculated to be 2.77 kg [40]. Based on the weight of the soil affected by the mushroom’s uptake, and on concentrations of the elements in the soil and bioavailable fraction, we calculated the amount of the elements in the bioavailable fraction (Table 1 and Table S6 in the Electronic Supplement).

Some features observed for elements such as Ag, Cd, and Pb are interesting, especially because Cd and Pb are toxic and dangerous for human health. Only insignificant amounts of Ag and Cd were present in the fruiting bodies of mushroom samples collected from most localities studied, which amounts could easily be taken by the mushrooms from the substrate (Tables 1 and 2, and Tables S3 and S6 in the Electronic Supplement). However, for samples collected from the granite-based FRD and peridotite-based SLP sites, the proportion of Ag and Cd in the mushrooms’ fruiting bodies can reach 50% of the elements’ amounts in the bioavailable fraction (Table S2 in the Electronic Supplement). There are no obvious reasons for mushrooms from these two particular sites to take so high amounts of Ag and Cd from the substrate while the amounts of the elements in the fruiting bodies were only 2–3% of the amounts present in the bioavailable fraction in all other cases (Table S2 in the Electronic Supplement). Therefore, it is reasonable to suggest that the excess of Ag and Cd in samples from the FRD and SLP sites was due to the incorporation of the elements from the air deposit, but not because of the direct uptake from the soil. The dominating wind directions through the whole month of August 2023 (the sampling month) were S-SW (Table S7 in the Electronic Supplement) over the FRD and SLP sites. Only shortly before sampling, the wind directions changed to N and NE for 2–3 days. Therefore, we can suggest that while the wind was blowing from the north and north-east, Ag- and Cd-bearing airborne particles (aerosols?) from a non-identified source might have been delivered to the FRD and SLP sites contaminating mushrooms directly. Such a suggestion about some element accumulation in the *I. badia* fruiting bodies being both site- and weather-related can reconcile contradictive data by [41] and [50].

Lead is often considered a monitoring element to estimate the degree of industrial pollution of the environment because high amounts of Pb were reported in mushrooms from areas affected by the pollution [e.g., 16, 17, 90, 91, 92]. However, although Pb is present both in the soils and in the bioavailable fractions in measurable amounts (in the bioavailable fraction, up to 8% of the concentration of the corresponding soil) in the case considered, the element’s concentrations in the mushrooms analyzed were always below the detection limit (Table 2 and Table S3 in the Electronic Supplement). Therefore, Pb was not the element readily taken by the *I. badia* from the soil (bioexcluded element). It can be said then that the lead’s uptake from the soils by the *I. badia* mushroom was insignificant (that is consistent with low mobility of the element in many mushroom species [e.g., 3, 19, 25, 41, 90], and contamination of soil by Pb would unlikely result in its high concentration in mushrooms fruiting bodies (at least in the case of the Boletaceae mushrooms). Therefore, the presence of high amounts of Pb in mushrooms’ fruiting bodies could rather point to contamination by airborne particles (aerosols?) enriched in Pb and resulted from recent industrial activity.

## Conclusions

The total content of 34 elements was determined by the ICP-OES measurements in samples of the *Imleria badia* mushroom and in corresponding growing substrate samples collected from six forested sites underlain by contrasting bedrock and affected differently by industrial pollution. Despite a limited data set, our research showed that the growing substrate element concentrations were only moderate to weak predictors for the elemental composition of the mushroom. Copper and Na were the elements for which site dependency was pronounced to the best. Part of the elements were taken by the mushroom from the bioavailable fraction, and part directly from the substrate. Additionally, a few elements (As, Cd, and Pb) could be accumulated from air deposits. In all cases, mushrooms behaved as bioconcentrating systems for elements such as Ag, K, P, Rb, S, and Se (BCF > 1) being, on the contrary, a bioexcluding system for the rest of the elements analyzed (BCF < 1). Most analyzed elements displayed moderate to high within-mushroom mobility accumulating preferably in the apical parts of the fruiting body (TF > 1). The highest within-mushroom mobility was demonstrated by Cd and Cu. Consumption of *I. badia* mushrooms from unpolluted or moderately polluted substrates does not seem to impose any risk for humans in terms of accumulation of heavy metals and other toxic elements. Overall, stipes accumulate much lesser amounts of toxic elements than the apical parts of the fruiting body do.

## Supplementary Information

Below is the link to the electronic supplementary material.

Supplementary file1 (DOC 35 KB)

Supplementary file2 (XLS 52 KB)

Supplementary file3 (DOC 89 KB)

Supplementary file4 (XLS 81 KB)

Supplementary file5 (DOC 67 KB)

Supplementary file6 (DOC 115 KB)

Supplementary file7 (XLS 73 KB)

Supplementary file8 (XLS 68 KB)

## Data Availability

The datasets obtained during the current study are available from the corresponding author upon reasonable request.
