# Supplementary material for: Effect of Compositionally Different Substrates on Elemental Properties of Bay Bolete Mushrooms: Case Study of 34 Essential and Non-essential Elements from Six Areas Affected Differently by Industrial Pollution
Source: Biol Trace Elem Res. 2024 Oct 31;203(7):3896–912. doi: 10.1007/s12011-024-04429-5 (PMC12174288; doi:10.1007/s12011-024-04429-5)
Supplement: Supplementary file 1 — Supplementary file1 (DOC 35 KB) [file 12011_2024_4429_MOESM1_ESM.doc]

**Materials and methods**

*Sampling and samples preparation*

We collected samples of *I. badia* in coniferous forests dominated by the Norway spruce (*Picea abies*). Availability of relevant literature (Antonín et al., 2019; Knauerová et al., 2020) and a free online mushroom atlas (<https://www.houbareni.cz/houby.php>) allowed us to identify mushroom species visually. Mushroom samples were added by substrate/soil samples collected from the depth down to 10-12 cm with the thin surface layer removed (see Borovička et al., 2019a). All samples were collected from both areas affected and not affected by the industrial pollution, and from each substrate type (granite-, amphibolite-, and peridotite-based). For the present study we changed the sampling strategy described in Andronikov et al. (2023a, b). Instead of collecting so-called mushroom-bearing and mushroom-free soil samples at each locality, we made a composite soil sample from several individual samples collected next to the mushroom sample on an area of ~1.5 x 1.5 m.

We applied sample preparation technique described in more details in, e.g., Dryżałowska & Falandysz (2014), Đurđić et al. (2021), Andronikov et al. (2023a, b). Briefly, mushroom samples were cleaned of all debris and shortly ultrasonicated in Milli-Q water. They were then separated into the stipe, the cap, and the sporophore subsamples, weighed and air-dried for several days. Thereafter, the subsamples were dried at 65ºC on a hot plate to constant weight (mean percentage of mushroom’s dry matter (DM) varied from 7.4±2.6% to 15.4±3.6%). About 0.5 g of each dried and pulverized in an gate mortar mushroom subsample were put into the pressure-resistant and analytical quality pro-digestive polytetrafluoroethylene (PTFE) vessel and pre-digested for 24 hours with concentrated HNO_3_ (65%; Ultrapure, Romil) at room temperature. Pre-digested subsamples were further digested under pressure in a MARS 6 (CEM Corp. Matthews, NC, USA) automatic microwave digestion system. The final HNO_3_-based digest was treated with concentrated Ultrapure Romil HCl and H_2_O_2_ and diluted to 0.3N HNO_3_ with Milli-Q water for further instrumental analysis. All sample solutions were filtered through an acid-cleaned syringe filter with a pore size of 0.45 μm before further processing.

Bulk substrate soil samples were first air dried for a few days and then sieved to a < 2 mm fraction. The sieved fraction of substrate samples has been ashed at 550°C for eight hours before processing and then homogenized and quantitatively dissolved in an HF–HClO_4_ mixture. The dissolved samples were dried down and diluted in a mixture of concentrated Ultrapure Romil HNO_3_ and Milli-Q water in order to get 50 mL of 0.3N HNO_3_-based sample solution.

To assess the mycoavailability of the elements in the soils, we conducted a single step extraction of mycoavailable fraction (Oulehle et al., 2010). Ten grams of the air dried and sieved to < 2 mm soil material were inserted in to the acid-cleaned 100 mL polypropelene bottle and 50 mL of 0.1M BaCl_2_ extraction solution were added. The samples were shaken with a multi-functional Orbital shaker PSU-20i for 2 hrs. Thereafter, 25 mL more of 0.1M BaCl_2_ extraction solution were added to each sample. Afterwards, the solutions were centrifuged at 5000 rpm for 10 min at the Megafuge ST Plus Series centrifuge and then filtered through 0.45 μm PTFE syringe filters. Filtrates were added with 25 mL 0.1 M BaCl_2_ extraction solution and with 1 mL of 6M HCl (up to 100 mL volume total).

*Analyses*

Elemental compositions of samples were determined with the the Agilent Technologies 5110 inductively coupled plasma optical emission spectrometer (ICP-OES) according to the standard operation procedure accepted by our laboratory (e.g., Andronikov et al., 2023a). The analytical procedure can be briefly described as follows. The samples in 0.3N HNO_3_ –based solution were introduced to plasma through the Meinhard concentric glass nebulizer via the Agilent Technologies SPS-4 autosampler. Samples of mycoavailable fraction were analyzed in 0.1 M BaCl_2_ extracts. During the analytical runs, ICP-OES was operated at the RF power of 1200 W, plasma Ar flow of 12 L min^-1^, auxiliary gas flow of 1.0 L min^-1^, and nebulizer gas flow of 0.7 L min^-1^. Altogether, 21 individual samples (seven fruiting bodies) of mushrooms and seven related soil samples (added with seven samples of mycoavailable fraction) were analyzed. The following 34 elements were measured for concentrations: Ag, Al, As, Ba, Ca, Cd, Co, Cr, Cu, Fe, Ga, K, Li, Mg, Mn, Mo, Na, Nb, Ni, P, Pb, Rb, S, Sb, Se, Sn, Sr, Ta, Ti, V, W, Y, Zn, and Zr. Sample concentrations were determined by first subtracting blank signal intensities from signal intensities obtained for the sample and standard solution.

A multi-element solution (ICP 23-element standard solution IV) from Merck KGaA (Germany) and mono-element standard solutions from Chromservis s.r.o. (Czech Republic) were used for preparation of standard solutions. The calibration for the analyzed elements was done for standard solution concentrations of 0.5, 1.0, and 2.0 mg L^-1^. A calibration curve was obtained by performing a linear least-squares regression for each element using the blank-subtracted counts and the known concentrations in each standard solution. The regression coefficients were 0.998 or higher in all cases. For quality control, we conducted repeated analyses of procedural blanks and standard reference materials (SRM) SRM NIST 1515 (apple leaves), SRM NIST 2709a (San Joaquin soil) and SRM NIST 2711a (Montana soil) in batches with unknown samples. Three replicate analyses per sample provided us with the analytical error below 1% RSD for all determinations. Both procedural blanks and SRMs were processed in the same way as unknown samples. In order to analyze Ag, As, Cd, Co, S, Sb, Sn, and W which concentrations were either too low in the SRM or data were not publicly available, solutions of known concentrations from mono-element solutions obtained from Chromservis s.r.o. (Czech Republic) were additionally prepared. Analytical results for the SRMs applied are given in the electronic supplement Table S1. The detection limits for the elements analyzed are given in electronic supplement Table S2. All sample preparation and analytical works were conducted in laboratories of the Czech Geological Survey (Prague).
