# Supplementary material for: Effect of Compositionally Different Substrates on Elemental Properties of Bay Bolete Mushrooms: Case Study of 34 Essential and Non-essential Elements from Six Areas Affected Differently by Industrial Pollution
Source: Biol Trace Elem Res. 2024 Oct 31;203(7):3896–912. doi: 10.1007/s12011-024-04429-5 (PMC12174288; doi:10.1007/s12011-024-04429-5)
Supplement: Supplementary file 3 — Supplementary file3 (DOC 89 KB) [file 12011_2024_4429_MOESM3_ESM.doc]

**Table S2** Response of different elements to extraction (%% with respect to the bulk soil)

|  | GRANITE-BASED | |  | AMPHIBOLITE-BASED | |
| --- | --- | --- | --- | --- | --- |
|  | FRD | SLG |  | JES | SLA |
| Ag |  |  |  |  |  |
| Al | 1.13% | 0.65% |  | 0.99% | 0.80% |
| As |  |  |  |  |  |
| Ba | n.d. | n.d. |  | n.d. | n.d. |
| Ca | 0.99% | 6.24% |  | 0.73% | 1.55% |
| Cd | 33.1% | 26.5% |  | 6.72% | 9.24% |
| Co | 0.53% | 0.76% |  | 0.90% | 3.00% |
| Cr | 0.11% | 1.58% |  | 0.07% | 0.03% |
| Cu | 19.5% | 23.7% |  | 7.08% | 1.59% |
| Fe | 0.54% | 0.25% |  | 0.05% | 0.06% |
| Ga |  |  |  |  |  |
| K | 0.40% | 0.50% |  | 0.84% | 2.36% |
| La | 2.20% | 5.58% |  | 2.74% | 1.86% |
| Li |  | 0.28% |  | 0.15% | 0.20% |
| Mg | 1.05% | 1.37% |  | 0.26% | 0.31% |
| Mn | 0.97% | 0.47% |  | 0.33% | 6.25% |
| Mo |  |  |  |  |  |
| Na | 0.61% | 0.20% |  | 0.58% | 0.50% |
| Nb |  |  |  |  |  |
| Ni | 5.12% | 9.79% |  | 0.85% | 1.19% |
| P | 0.18% | 1.11% |  | 0.12% | 0.11% |
| Pb | 9.47% | 30.1% |  | 6.09% | 4.17% |
| Rb | 0.03% | 0.33% |  | 0.07% | 0.21% |
| S | 1.46% | 3.21% |  | 1.27% | 1.61% |
| Sb |  |  |  |  |  |
| Se |  |  |  |  |  |
| Sn |  |  |  |  |  |
| Sr | 0.42% | 5.93% |  | 0.49% | 2.54% |
| Ta |  |  |  |  |  |
| Ti | 0.002% | 0.004% |  | 0.002% | 0.002% |
| V | 1.38% | 1.48% |  | 0.54% | 0.81% |
| W | 6.42% | 1.48% |  |  |  |
| Y | 0.88% | 5.41% |  | 1.51% | 0.58% |
| Zn | 5.36% | 3.68% |  | 1.82% | 2.28% |
| Zr |  |  |  |  |  |

**Table 1** (continued)

|  |  | PERIDOTITE-BASED |  |
| --- | --- | --- | --- |
|  | RAN | RAN* | SLP |
| Ag |  |  |  |
| Al | 0.08% | 0.20% | 0.58% |
| As |  |  |  |
| Ba | n.d. | n.d. | n.d. |
| Ca | 10.2% | 10.6% | 2.54% |
| Cd | 21.6% | 4.40% | 11.7% |
| Co | 0.52% | 2.15% | 4.74% |
| Cr | 0.02% | 0.01% | 0.10% |
| Cu | 8.01% | 1.22% | 7.81% |
| Fe | 0.01% | 0.002% | 0.21% |
| Ga |  |  |  |
| K | 0.51% | 2.82% | 2.23% |
| La | 0.68% | 3.87% | 1.34% |
| Li | 0.01% | 0.58% | 0.23% |
| Mg | 17.0% | 10.9% | 1.11% |
| Mn | 3.10% | 4.42% | 8.52% |
| Mo |  |  |  |
| Na | 0.55% | 1.46% | 0.63% |
| Nb |  |  |  |
| Ni | 9.70% | 2.62% | 4.18% |
| P | 0.20% | 0.13% | 0.92% |
| Pb | 0.72% | 3.57% | 7.79% |
| Rb | 0.29% | 0.31% | 0.04% |
| S | 0.54% | 0.92% | 3.02% |
| Sb |  |  |  |
| Se |  |  |  |
| Sn |  |  |  |
| Sr | 2.01% | 9.79% | 2.70% |
| Ta |  |  |  |
| Ti |  |  |  |
| V | 0.29% | 0.03% | 0.07% |
| W |  |  |  |
| Y | 0.45% | 3.46% | 0.75% |
| Zn | 5.00% | 3.07% | 9.69% |
| Zr |  |  |  |

Note: n.d., no data available
