# Supplementary material for: Effect of Compositionally Different Substrates on Elemental Properties of Bay Bolete Mushrooms: Case Study of 34 Essential and Non-essential Elements from Six Areas Affected Differently by Industrial Pollution
Source: Biol Trace Elem Res. 2024 Oct 31;203(7):3896–912. doi: 10.1007/s12011-024-04429-5 (PMC12174288; doi:10.1007/s12011-024-04429-5)
Supplement: Supplementary file 5 — Supplementary file5 (DOC 67 KB) [file 12011_2024_4429_MOESM5_ESM.doc]

**Table S4** Bioconcentration factor for the studied *I. badia* samples (bulk fruiting body/soil)

|  | Granite-based | |  | Amphibolite-based | |  |  | Peridotite-based | |
| --- | --- | --- | --- | --- | --- | --- | --- | --- | --- |
|  | FRD | SLG |  | JES | SLA |  | RAN | RAN* | SLP |
|  |  |  |  |  |  |  |  |  |  |
| Ag | 6.65 | 0.855 |  | 2.24 | 1.23 |  | 3.47 | 6.69 | 4.72 |
| Al | <0.001 | <0.001 |  | 0.001 | 0.001 |  | <0.001 | <0.001 | 0.001 |
| As | 0.038 | 0.139 |  | 0.050 | 0.037 |  | 0.014 | 0.128 | 0.160 |
| Ba | 0.002 | 0.006 |  | 0.003 | 0.004 |  | <0.001 | 0.002 | 0.002 |
| Ca | 0.083 | 0.149 |  | 0.023 | 0.037 |  | 0.068 | 0.037 | 0.028 |
| Cd | 47.0 | 18.8 |  | 0.396 | 0.310 |  | 1.86 | 0.398 | 7.45 |
| Co | n.d. | n.d. |  | n.d. | n.d. |  | n.d. | n.d. | n.d. |
| Cr | 0.026 | 0.028 |  | 0.003 | 0.002 |  | 0.001 | <0.001 | 0.001 |
| Cu | 6.75 | 6.49 |  | 1.50 | 0.660 |  | 1.88 | 0.490 | 3.14 |
| Fe | 0.002 | 0.004 |  | 0.001 | 0.001 |  | 0.001 | <0.001 | 0.001 |
| Ga | n.d. | n.d. |  | n.d. | n.d. |  | n.d. | n.d. | n.d. |
| K | 1.06 | 1.01 |  | 2.22 | 2.33 |  | 1.45 | 5.29 | 1.81 |
| Li | n.d. | n.d. |  | n.d. | n.d. |  | n.d. | n.d. | n.d. |
| Mg | 0.425 | 0.977 |  | 0.062 | 0.039 |  | 0.079 | 0.040 | 0.035 |
| Mn | 0.041 | 0.064 |  | 0.021 | 0.032 |  | 0.002 | 0.004 | 0.015 |
| Mo | 0.284 | n.d. |  | n.d. | n.d. |  | n.d. | n.d. | n.d. |
| Na | 0.149 | 0.164 |  | 0.167 | 0.157 |  | 0.198 | 0.408 | 0.178 |
| Nb | 0.002 | 0.007 |  | 0.003 | 0.007 |  | 0.008 | 0.013 | n.d. |
| Ni | 0.089 | 0.286 |  | 0.019 | 0.015 |  | 0.003 | 0.001 | 0.016 |
| P | 7.50 | 14.7 |  | 8.41 | 9.68 |  | 11.8 | 12.4 | 27.4 |
| Pb | n.d. | n.d. |  | n.d. | n.d. |  | n.d. | n.d. | n.d. |
| Rb | 4.21 | 3.51 |  | 6.40 | 7.64 |  | 3.38 | 5.26 | 4.78 |
| S | 17.6 | 24.8 |  | 10.7 | 10.6 |  | 17.8 | 16.8 | 29.1 |
| Sb | n.d. | n.d. |  | n.d. | n.d. |  | n.d. | n.d. | n.d. |
| Se | n.d. | n.d. |  | n.d. | n.d. |  | n.d. | n.d. | n.d. |
| Sn | 0.054 | 0.014 |  | n.d. | n.d. |  | n.d. | 0.253 | n.d. |
| Sr | 0.012 | 0.030 |  | 0.010 | 0.018 |  | 0.003 | 0.015 | 0.012 |
| Ta | n.d. | n.d. |  | n.d. | n.d. |  | n.d. | n.d. | n.d. |
| Ti | <0.001 | <0.001 |  | <0.001 | <0.001 |  | <0.001 | <0.001 | <0.001 |
| V | n.d. | n.d. |  | 0.002 | n.d. |  | n.d. | n.d. | n.d. |
| W | 0.167 | 0.102 |  | 0.740 | 0.501 |  | 0.333 | 0.861 | 0.747 |
| Y | n.d. | n.d. |  | n.d. | n.d. |  | n.d. | n.d. | n.d. |
| Zn | 7.34 | 2.92 |  | 1.52 | 0.900 |  | 0.994 | 1.01 | 1.22 |
| Zr | n.d. | n.d. |  | n.d. | n.d. |  | n.d. | n.d. | n.d. |
