# Supplementary material for: Effect of Compositionally Different Substrates on Elemental Properties of Bay Bolete Mushrooms: Case Study of 34 Essential and Non-essential Elements from Six Areas Affected Differently by Industrial Pollution
Source: Biol Trace Elem Res. 2024 Oct 31;203(7):3896–912. doi: 10.1007/s12011-024-04429-5 (PMC12174288; doi:10.1007/s12011-024-04429-5)
Supplement: Supplementary file 6 — Supplementary file6 (DOC 115 KB) [file 12011_2024_4429_MOESM6_ESM.doc]

**Table S5** Translocation factor for the studied *I. badia* samples

|  |  | GRANITE-BASED | |  |  |  | AMPHIBOLITE-BASED | | |
| --- | --- | --- | --- | --- | --- | --- | --- | --- | --- |
|  | FRD | FRD | SLG | SLG |  | JES | JES | SLA | SLA |
|  | Cap/Stp | Spr/Cap | Cap/Stp | Spr/Cap |  | Cap/Stp | Spr/Cap | Cap/Stp | Spr/Cap |
|  |  |  |  |  |  |  |  |  |  |
| Ag | 1.85 | 1.41 | 1.12 | 1.23 |  | 2.40 | 3.81 | 1.09 | 1.05 |
| Al | 1.09 | 1.65 | 1.52 | 1.20 |  | 1.83 | 0.90 | 1.15 | 1.90 |
| As | 1.29 | 1.45 | 1.97 | 1.15 |  | 2.38 | 1.04 | 1.70 | 1.14 |
| Ba | 1.03 | 1.50 | 1.34 | 1.31 |  | 1.54 | 0.54 | 0.52 | 1.76 |
| Ca | 2.79 | 0.91 | 3.46 | 0.74 |  | 1.53 | 0.58 | 1.38 | 0.74 |
| Cd | 1.24 | 4.32 | 1.55 | 2.95 |  | 1.92 | 2.96 | 1.59 | 1.30 |
| Co | n.d. | n.d. | n.d. | n.d. |  | n.d. | n.d. | n.d. | n.d. |
| Cr | 0.76 | 8.01 | 1.08 | 2.07 |  | 1.51 | 2.06 | 2.21 | 0.77 |
| Cu | 1.37 | 3.67 | 1.11 | 2.68 |  | 1.56 | 3.79 | 1.65 | 1.53 |
| Fe | 1.52 | 2.37 | 1.21 | 1.98 |  | 1.65 | 1.40 | 1.30 | 1.53 |
| Ga | n.d. | n.d. | n.d. | n.d. |  | n.d. | n.d. | n.d. | n.d. |
| K | 1.22 | 0.47 | 1.50 | 0.43 |  | 1.16 | 0.43 | 1.25 | 0.91 |
| Li | n.d. | n.d. | n.d. | n.d. |  | n.d. | n.d. | n.d. | n.d. |
| Mg | 1.47 | 2.05 | 1.26 | 1.95 |  | 1.66 | 1.99 | 1.63 | 0.90 |
| Mn | 1.39 | 1.51 | 0.83 | 1.78 |  | 1.03 | 0.82 | 0.37 | 2.09 |
| Mo | 1.83 | 1.45 | 1.17 | 1.87 |  | 1.18 | 1.62 | 1.67 | 1.13 |
| Na | 0.46 | 0.47 | 0.93 | 0.58 |  | 0.60 | 0.44 | 0.85 | 0.73 |
| Nb | 0.31 | 0.91 | 0.36 | 0.39 |  | 0.40 | 1.00 | 2.16 | 1.00 |
| Ni | 1.89 | 1.56 | 1.33 | 1.76 |  | 0.78 | 1.86 | 2.43 | 0.72 |
| P | 1.39 | 2.83 | 1.35 | 2.28 |  | 1.86 | 3.38 | 1.64 | 1.02 |
| Pb | n.d. | n.d. | n.d. | n.d. |  | n.d. | n.d. | n.d. | n.d. |
| Rb | 1.57 | 0.61 | 1.69 | 0.52 |  | 1.57 | 0.53 | 1.51 | 0.59 |
| S | 1.42 | 1.58 | 1.41 | 1.72 |  | 2.33 | 1.75 | 2.68 | 0.61 |
| Sb | n.d. | n.d. | n.d. | n.d. |  | n.d. | n.d. | n.d. | n.d. |
| Se | 1.76 | 0.57 | 1.75 | 0.62 |  | 1.67 | 0.75 | 1.54 | 0.70 |
| Sn | 0.69 | 1.28 | 1.01 | 0.56 |  | 1.13 | 0.84 | 0.91 | 1.14 |
| Sr | 1.39 | 1.37 | 2.23 | 1.18 |  | 1.30 | 0.72 | 0.69 | 1.37 |
| Ta | n.d. | n.d. | n.d. | n.d. |  | n.d. | n.d. | n.d. | n.d. |
| Ti | 2.74 | 1.15 | 0.70 | 2.21 |  | 1.86 | 0.92 | 1.38 | 1.59 |
| V | n.d. | n.d. | n.d. | n.d. |  | 2.14 | 0.80 | n.d. | n.d. |
| W | 1.09 | 1.46 | 1.30 | 1.40 |  | 1.38 | 1.85 | 1.08 | 1.08 |
| Y | n.d. | n.d. | n.d. | n.d. |  | n.d. | n.d. | n.d. | n.d. |
| Zn | 1.41 | 1.50 | 1.06 | 1.49 |  | 1.47 | 1.65 | 1.13 | 1.04 |
| Zr | n.d. | n.d. | n.d. | n.d. |  | n.d. | n.d. | n.d. | n.d. |

**Table 3** (continued)

|  |  |  | PERIDOTITE-BASED | |  |  |
| --- | --- | --- | --- | --- | --- | --- |
|  | RAN | RAN | RAN* | RAN* | SLP | SLP |
|  | Cap/Stp | Spr/Cap | Cap/Stp | Spr/Cap | Cap/Stp | Spr/Cap |
|  |  |  |  |  |  |  |
| Ag | 2.91 | 1.92 | 2.14 | 2.20 | 1.23 | 1.30 |
| Al | 0.78 | 2.02 | 2.05 | 0.87 | 1.04 | 1.03 |
| As | 1.08 | 1.07 | 1.43 | 1.42 | 1.26 | 1.02 |
| Ba | 0.47 | 1.42 | 1.04 | 0.72 | 1.33 | 0.88 |
| Ca | 2.05 | 0.64 | 1.28 | 0.90 | 1.33 | 0.77 |
| Cd | 1.87 | 2.04 | 1.40 | 3.30 | 1.71 | 1.85 |
| Co | n.d. | n.d. | n.d. | n.d. | 0.72 | 0.71 |
| Cr | 1.17 | 1.69 | 1.64 | 1.56 | 1.20 | 0.88 |
| Cu | 1.78 | 1.93 | 1.41 | 3.40 | 1.84 | 1.07 |
| Fe | 1.25 | 1.67 | 1.65 | 1.48 | 1.45 | 1.31 |
| Ga | n.d. | n.d. | n.d. | n.d. | n.d. | n.d. |
| K | 1.64 | 0.77 | 1.14 | 0.44 | 1.44 | 0.87 |
| Li | n.d. | n.d. | n.d. | n.d. | n.d. | n.d. |
| Mg | 1.40 | 1.58 | 1.10 | 2.56 | 1.49 | 0.98 |
| Mn | 0.72 | 1.56 | 1.50 | 0.96 | 1.24 | 1.27 |
| Mo | 1.18 | 1.65 | 1.17 | 1.74 | n.d. | n.d. |
| Na | 0.28 | 0.95 | 1.14 | 0.41 | 0.50 | 0.69 |
| Nb | 1.00 | 3.21 | 1.12 | 0.18 | 1.32 | n.d. |
| Ni | 0.88 | 1.94 | 1.14 | 1.73 | 0.99 | 0.89 |
| P | 1.84 | 1.71 | 1.70 | 3.15 | 1.51 | 1.06 |
| Pb | n.d. | n.d. | n.d. | n.d. | n.d. | n.d. |
| Rb | 1.05 | 0.96 | 1.29 | 0.60 | 0.75 | 0.93 |
| S | 1.48 | 1.48 | 1.76 | 1.45 | 0.87 | 0.88 |
| Sb | n.d. | n.d. | n.d. | n.d. | n.d. | n.d. |
| Se | 1.34 | 0.91 | 2.51 | 0.57 | 1.30 | 0.95 |
| Sn | 1.09 | 1.09 | 0.90 | 0.95 | 0.95 | 1.09 |
| Sr | 0.68 | 1.38 | 1.16 | 0.98 | 1.06 | 0.86 |
| Ta | n.d. | n.d. | n.d. | n.d. | n.d. | n.d. |
| Ti | 0.51 | 2.11 | 2.32 | 0.44 | 0.91 | 1.20 |
| V | n.d. | n.d. | n.d. | n.d. | n.d. | n.d. |
| W | 1.50 | 1.13 | 1.43 | 1.13 | 1.31 | 1.11 |
| Y | n.d. | n.d. | n.d. | n.d. | n.d. | n.d. |
| Zn | 1.18 | 1.26 | 1.32 | 1.19 | 1.34 | 1.15 |
| Zr | n.d. | n.d. | n.d. | n.d. | n.d. | n.d. |

Stp, stipe; Spr, sporophore
